# Sex-based differences in patients with locally advanced pharyngeal and laryngeal SCC treated with definitive or adjuvant radiotherapy

**DOI:** 10.1186/s13014-025-02771-z

**Published:** 2025-12-04

**Authors:** Linda Agolli, Luise Reinhard, Ahmed Gawish, Christine Langer, Christoph Arens, Gabriele A. Krombach, Sebastian Harth, Leon Wendrich, Ann-Katrin Exeli, Stefan Gattenlöhner, Sebastian Adeberg, Daniel Habermehl

**Affiliations:** 1 Department of Radiation Oncology, Justus-Liebig-University Giessen, Giessen-Marburg University Hospital, 35392 Giessen, Germany; 2 Department of Radiotherapy and Radiation Oncology, Philipps-University Marburg, Marburg University Hospital, Marburg, Germany; 3 Marburg Ion-Beam Therapy Center (MIT), Department of Radiotherapy and Radiation Oncology, Marburg University Hospital, Marburg, Germany; 4 Department of Otorhinolaryngology, Justus-Liebig-University Giessen, Giessen University Hospital, Giessen, Germany; 5 Department of Diagnostic and Interventional Radiology, Justus-Liebig-University Giessen, Giessen University Hospital , Giessen, Germany; 6 Institute of Pathology, Justus-Liebig-University Giessen, Giessen University Hospital, Giessen, Germany

**Keywords:** Sex-differences, Head and neck cancer, Outcome, Toxicity, Radiotherapy

## Abstract

**Aim:**

To evaluate sex-based differences in survival outcomes, toxicity, and patterns of local recurrence in patients with locally advanced head and neck squamous cell carcinoma (HNSCC) treated with definitive or adjuvant radiotherapy (RT).

**Methods:**

We conducted a retrospective review of 309 patients (246 males, 63 females) diagnosed with primary squamous cell carcinoma of the oropharynx, larynx, or hypopharynx treated with curative-intent RT at our institution between 2016 and 2023. Inclusion criteria comprised histologically confirmed SCC, stage T3/T4 and/or node-positive disease, and complete RT treatment with adequate follow-up. Survival endpoints—overall survival (OS), progression-free survival (PFS), and metastasis-free survival (MFS)—were analyzed using the Kaplan-Meier method and log-rank tests. Patterns of local failure were classified using an established five-type system: A (central high-dose), B (peripheral high-dose), C (central intermediate- or low-dose), D (peripheral intermediate- or low-dose), and E (extraneous dose). Treatment-related toxicity was also compared between sexes.

**Results:**

No significant differences in OS, PFS, or MFS were found between male and female patients across all treatment subgroups. Log rank test did not identify any significant prognostic factor for survival and local recurrence. However, female patients experienced a higher rate of grade ≥ 3 dermatitis (12.7% vs. 5.3%, *p* = 0.037). Pattern A nodal failures (central high-dose volume) were significantly more common in females (64.3%) than in males (28.8%; *p* = 0.014), while other recurrence patterns showed no significant sex-based differences.

**Conclusion:**

Sex was not an independent predictor of survival in this cohort of locally advanced HNSCC patients. Nevertheless, the higher rate of severe skin toxicity and nodal failures in females highlights a potential need for sex-adapted radiotherapy strategies and further prospective investigation.

**Clinical trial number:**

Not applicable.

**Supplementary Information:**

The online version contains supplementary material available at 10.1186/s13014-025-02771-z.

## Introduction

Sex disparities encompass multifactorial elements, such as physiology, genetics, and environment in various human diseases including cancer sometimes due to sex-specific gene expression during critical developmental stages and gene editing processes [1]. Squamous cell carcinoma (SCC) of the pharynx and larynx constitutes a significant subset of head and neck cancers (HNC), with definitive or postoperative radiotherapy (RT) or chemoradiation (CRT) being a cornerstone of treatment [2]. While the incidence of HNC is higher in men, emerging evidence indicates notable sex-based differences in tumor biology, treatment responses, and survival outcomes, particularly in locally advanced stages [3].

Epidemiological data reveal that men are disproportionately affected by laryngeal and hypopharyngeal cancers, with incidence rates up to 20 times higher than in women, even after adjusting for risk factors like smoking and alcohol consumption [3]. However, women often present with more advanced-stage disease and are more likely to have supraglottic tumors [4]. Despite this, several studies have reported better overall survival (OS) and disease-specific survival (DSS) in women with laryngeal SCC, potentially due to factors such as lower tobacco exposure and higher rates of smoking cessation post-diagnosis [4].

In the context of oropharyngeal SCC, sex differences are further influenced by human papillomavirus (HPV) status. Women with HPV-negative tumors have been shown to experience poorer survival outcomes compared to men, a disparity that persists even after adjusting for treatment modalities [5]. Additionally, treatment delays, particularly in initiating adjuvant radiotherapy, have been more commonly observed in women and are associated with worse prognoses [6].

Despite these observed disparities, some studies have found no significant sex-based differences in treatment strategies or survival outcomes when patients receive similar multidisciplinary care [7]. These conflicting findings underscore the complex interplay of sex-related biological, behavioral, and healthcare access factors in head and neck cancer, emphasizing the urgent need for more comprehensive, sex-specific research to inform tailored treatment strategies and improve patient outcomes [8].

Large studies investigating sex differences in factors impacting prognosis and sex-specific outcome in patients with head and neck cancer treated with radiotherapy are insufficient. This study aims to investigate sex differences in patients with locally advanced pharyngeal and laryngeal SCC treated with definitive or adjuvant radiotherapy. We evaluated treatment patterns, prognostic factors and survival outcomes, and also the impact of sex on disease progression and toxicity in order to explore more personalized and equitable treatment approaches.

## Materials and methods

### Patient selection

We conducted a retrospective review of medical records for patients diagnosed with primary SCC of the oropharynx, larynx, or hypopharynx who received either definitive or postoperative RT at our Department of Radiation Oncology between 2016 and 2023. Inclusion criteria were as follows: histologically confirmed SCC, locally advanced disease (defined as clinical T3/T4 and/or node-positive, N+), curative-intent RT, completed RT at the prescribed dose, and sufficient documentation on surgical intervention, systemic therapy, and follow-up. Exclusion criteria included the presence of distant metastases at the time of RT initiation, non-SCC histology, tumors of the oral cavity or lateralized subsites, interruptions in RT or receipt of palliative RT dosing.

Patient data, including clinical records, imaging, and follow-up outcomes, were extracted from electronic medical records. A total of 309 patients met the inclusion criteria: 246 males and 63 females. All patients received either definitive or adjuvant RT, with or without concurrent systemic therapy.

### Definition of sex

Sex was defined as a biological variable, based on the sex assigned at birth (male or female) as recorded in the medical records. Gender identity was not assessed due to the retrospective design of the study and the absence of consistent documentation in the clinical data. This approach is in line with recommendations for sex-based analyses in biomedical research, which emphasize the importance of distinguishing sex (a biological construct) from gender (a sociocultural construct), particularly in retrospective analyses where gender data are often unavailable or unreliable [9].

### Treatment

All patients underwent simulation with planning computed tomography (CT) under thermoplastic mask immobilization. The gross tumor volume (GTV) included the primary tumor and any radiologically or clinically involved lymph nodes. Two clinical target volumes (CTVs) were defined: the high-risk CTV (HR-CTV), encompassing the macroscopic tumor and/or postoperative tumor bed and involved nodal regions; and the low-risk CTV (LR-CTV), covering elective nodal levels, delineated according to institutional selective neck irradiation protocols. Radiotherapy was delivered in 28–33 fractions.

In the definitive setting, the HR-CTV received a median dose of 70.2 Gy (range: 56–72 Gy), and the LR-CTV a median of 57.6 Gy (range: 50.4–57.6 Gy). In the postoperative setting, the HR-CTV received a median dose of 66 Gy (range: 59.4–67.5 Gy), and the LR-CTV 54 Gy (range: 50.4–54 Gy). Planning target volumes (PTVs) were generated by applying a 3–5 mm margin to each CTV to compensate for setup variability.

Organs at risk (OARs), including the parotid and submandibular glands, mandible, pharynx, oral cavity, larynx, spinal cord, brainstem, optic chiasm, and optic nerves, were contoured for dose constraints. The same target delineation and dose prescription guidelines were applied regardless of HPV status according to the discretion of the treating radiation oncologist [10, 11].

All radiotherapy plans were generated using either intensity-modulated radiotherapy (IMRT) or volumetric modulated arc therapy (VMAT) with 6 MV photons. Image guidance was performed with cone-beam CT (CBCT), typically once or twice per week, or daily as needed for treatment verification.

Concurrent systemic therapy was administered according to standard institutional protocols and based on treatment intent, performance status, comorbidities, and tumor characteristics. In the definitive setting, most patients received concurrent chemoradiotherapy with weekly cisplatin (40 mg/m²), with alternative regimens such as carboplatin/paclitaxel, or immunotherapy with cetuximab in patients deemed cisplatin-ineligible.

In the postoperative setting, systemic therapy was generally reserved for patients with high-risk pathological features, including extranodal extension or positive margins. Weekly cisplatin was most commonly used in this group as well, though a larger proportion of patients were treated with radiotherapy alone due to age or comorbidities. All systemic therapy decisions were made by a multidisciplinary tumor board. The type of agent, number of cycles, and timing were recorded and included in the analysis.

### Outcomes and statistical analysis

Post-treatment follow-up included imaging with MRI, contrast-enhanced CT, or PET-CT, as clinically indicated. All suspected recurrences were reviewed by board-certified radiologists specialized in head and neck oncology.

This study aimed to assess sex-related differences in survival, disease control, and treatment-related toxicity between female and male patients. Baseline patient and tumor characteristics were compared between sexes. Additionally, we analyzed the potential prognostic impact of clinical factors—including comorbidities, Karnofsky Performance Status (KPS), HPV and p16-status, T- and N-classification, systemic therapy, and the occurrence of recurrence or metastasis on survival outcomes stratified by sex.

Overall survival (OS) was defined as the interval from the last day of radiotherapy to death from any cause or last follow-up. Progression-free survival (PFS) was defined as the time from completion of RT to the occurrence of locoregional or distant progression, or death. Metastasis-free survival (MFS) was calculated from the end of RT to the development of distant metastases, death, or last follow-up.

Patterns of locoregional recurrence were categorized based on the methodology by Mohamed et al. [12], as follows: pattern A (central high-dose), pattern B (peripheral high-dose), pattern C (central elective dose), pattern D (peripheral elective dose), and pattern E (extraneous dose).

Toxicity was assessed retrospectively using clinical documentation and graded according to the Common Terminology Criteria for Adverse Events (CTCAE), version 5.0.

For qualitative variables, absolute and percentage frequencies were provided. Gender was compared using contingency tables and tested for association with the chi-square test. If the expected frequencies were too low, Fisher’s exact test was applied instead. Furthermore, Kaplan-Meier survival analyses were performed and the influence of gender was tested using the log-rank test. All statistical tests were two-sided at a significance level of 5%.

Due to the descriptive nature of the present analysis, no alpha adjustment for multiple testing was applied and the results were interpreted accordingly. The statistical analyses were performed using IBM SPSS Statistics 30 (SPSS Inc., an IBM company, Chicago, IL).

### Ethics

The study was performed in accordance with the Declaration of Helsinki Version 2013. All the patients signed the informed consent and the study was approved from our internal review board of Justus-Liebig University of Giessen (ethic approval AZ 73/24).

## Results

### Patients´ characteristics

Among the 309 patients included in the analysis, 63 (20.4%) were female and 246 (79.6%) males. The median age at diagnosis was similar between females (62 years) and males (65 years; *p* = 0.514). No significant sex differences were observed in performance status, smoking or alcohol use, or the prevalence of major comorbidities, although other lung diseases were significantly more common in females (11.1%) than in males (1.6%; *p* = 0.002). Overall, 177 patients (57.3%) received definitive radiotherapy (RT), and a total of 132 (42.7%) patients underwent adjuvant RT. The distribution of treatment modality did not differ significantly between sexes (*p* = 0.161). In the adjuvant RT setting, the median age was 61 years for females and 62 years for males, while in the definitive RT group, females had a median age of 63 years compared to 68 years in males; neither difference was statistically significant. These results are summarized in Table 1.

**Table 1:** Sex-Based differences in Patient, characteristics among patients receiving adjuvant or definitive radiotherapy (*n* = 309)

Variable	Female (*n* = 63; 100%)	Male (*n* = 246; 100%)	*p*-value
**Median age at diagnosis**	62	65	0.514
- Range	29–84	40–89	
**Karnofsky-Index**			
- 90–100%	31 (49.2%)	137 (55.7%)	0.405
- 80 − 70%	32 (50.8%)	108 (43.9)	
- 60%	0 (0%)	1 (0.4%)	
**Smoke**			
- Never	9 (19.1%)	34 (19.2%)	0.445
- Quit	7 (14.9%)	41 (23.2%)	
- Current	31 (66.0%)	102 (57.6%)	
- Missing			
**Alcohol**			
- yes	19 (30.2%)	97 (39.4%)	0.085
- no	14 (22.2%)	36 (14.6%)	
- missing	30 (47.6%)	113 (45.9%)	
**Comorbidity**			
- COPD	6 (9.5%)	17 (6.9%)	0.433
- Other lung disease	7 (11.1%)	4 (1.6%)	**0.002**
- Heart disease	11 (17.5%)	56 (22.8%)	0.362
- Arterial hypertension	34 (54%)	116 (47.2%)	0.334
- Diabetes	6 (9.5%)	41 (16.7%)	0.159
- Other malignacies	10 (15.9%)	21 (8.5%)	*0.084*
- Stroke	5 (7.9%)	15 (6.1%)	0.572

The oropharynx was the most frequent primary tumor site in both sexes (73% in females vs. 63% in males; *p* = 0.277), particularly in the adjuvant RT cohort (86.4% of females vs. 62.7% of males; *p* = 0.068). HPV positivity was diagnosed in 66.7% of patients in both groups. Among those receiving adjuvant RT, HPV-positive tumors were slightly more common in females (59.1%) than males (36.4%; *p* = 0.047), and p16-positivity was significantly higher in females (59.1%) than males (39.1%; *p* = 0.017). Tumor grade distribution was similar, with no histologically grade 1 tumors found in females. T-stage and N-stage distributions showed no significant sex differences overall or by treatment group.

In the adjuvant RT cohort, R0 resection rates and the presence of extracapsular extension (ECE) in lymph nodes were comparable between males and females (*p* > 0.999, respectively).

There were no significant differences in systemic therapy use between sexes (female: 60.3% vs. male: 64.6%, *p* = 0.461). However, systemic treatment was clearly more prevalent in the definitive setting. These results are summarized in Table 2.

**Table 2:** Sex-Based differences in tumor and treatment characteristics among patients receiving adjuvant or definitive radiotherapy

Variable	Overall			Adjuvant RT (*n* = 132)			Definitive RT (*n* = 177)		
	Female	Male	*p*-value	Female	Male	*p*-value	Female	Male	*p*-value
	*63 (100%)*	*246 (100%)*		*22 (34.9%)*	*110 (44.7%)*		*41 (65.1%)*	*136 (55.3%)*	0.161
**Tumor site**									
- oropharynx	46 (73%)	155 (63%)	0.277	19 (86.4%)	69 (62.7%)	*0.068*	27 (65.9%)	86 (63.2%)	0.577
- hypopharynx	9 (14.3%)	39 (15.9%)		0 (0.0%)	15 (13.6%)		9 (22.0%)	24 (17.6%)	
- larynx	8 (12.7%)	52 (21.1%)		3 (13.6%)	26 (23.6%)		5 (12.2%)	26 (19.1%)	
**HPV-status**									
- positive	42 (66.7%)	164 (66.7%)	> 0,999	13 (59.1%)	40 (36.4%)	**0.047**	8 (19.5%)	42 (30.9%)	0.156
- negative	21 (33.3%)	82 (33.3%)		9 (40.9%)	70 (63.6%)		33 (80.5%)	94 (69.1%)	
**p-16 positive**	20 (31.7%)	54 (22%)	0.108	13 (59.1%)	35 (39.1%)	**0.017**	7 (17.1%)	19 (14%)	0.623
**Grade**									
- grade 1	0 (0.0%)	4 (1.6%)	0.860	0 (0.0%)	2 (1.8%)	1.000	0 (0.0%)	2 (1.5%)	1.000
- grade 2	35 (57.4%)	134 (55.1%)		12 (54.5%)	58 (53.2%)		23 (59.0%)	76 (56.7%)	
- grade 3	26 (42.6%)	105 (43.2%)		10 (45.5%)	49 (45.0%)		16 (41.0%)	56 (41.8%)	
**T-Stage**									
- T1	15 (23,8%)	26 (10,6%)	0.271	11 (50.0%)	19 (17.3%)	*0.052*	4 (9.8%)	7 (5.1%)	0.696
- T2	12 (19,0%)	72 (29,3%)		4 (18.2%)	43 (39.1%)		8 (19.5%)	29 (21.3%)	
- T3	17 (27%)	74 (30.1%)		3 (13.6%)	24 (21.8%)		14 (34.1%)	50 (36.8%)	
- T4	19 (30.1%)	74 (30.1%)		4 (18.2%)	24 (21.8%)		15 (36.6%)	50 (36.8%)	
**N-Stage**									
- N0	12 (19%)	64 (26%)	0.649	5 (22.7%)	22 (20.0%)	0.891	12 (19.0%)	64 (26.0%)	0.126
- N1	19 (30.2%)	59 (24%)		9 (40.9%)	45 (40.9%)		19 (30.25)	59 (24.0%)	
- N2	25 (39.7%)	96 (39%)		4 (18.2%)	27 (24.5%)		25 (39.7%)	96 (39.0%)	
- N3	7 (11.1%)	23 (9.3%)		4 (18.2%)	14 (12.7%)		7 (11.1%)	23 (9.3%)	
- Nx	0 (0%)	4 (1.6%)		0 (0.0%)	2 (1.2%)		0 (0.0%)	4 (1.6%)	
**R-status after OP**									
- R0				17 (77.3%)	81 (73.6%)	> 0,999			
- R1				3 (13.6%)	16 (14.5%)				
- Rx				2 (9.1%)	13 (11.8%)				
**Lymph nodes with ECE after OP**				6 (27.3%)	30 (27.3%)	> 0,999			
**Concurrent systemic therapy**									0.746
- Yes - no	38 (60.3%) 25 (39.7%)	161 (64.6%) 87 (35.4%)	0.461	6 (27.3%) 16 (72.7%)	47 (42.7%) 63 (57.3%)	0.131	32 (78.0%) 9 (22.0%)	112 (82.4%) 24 (17.6%)	
- Cisplatin weekly	25 (39.7%)	111 (45.1%)		4 (18.2%)	42 (38.2%)		21 (51.2%)	69 (50.7%)	
- Other chemotherapy schema	13 (20.6%)	48 (19.5%)		2 (9.1%)	5 (4.5%)		11 (26.8%)	43 (31.7%)	
- Cetuximab	6 (9.5%)	11 (4.5%)	0.126				6 (14.6%)	11 (8.1%)	0.231

### Toxicity

Overall treatment-related toxicity was common and generally comparable between female and male patients. The most frequent toxicities in both groups were fatigue (73.0% in females vs. 75.2% in males, *p* = 0.721), dysphagia (85.7% vs. 79.7%, *p* = 0.276), and dermatitis (95.2% vs. 91.1%, *p* = 0.278). Grade ≥ 3 dermatitis occurred more frequently in females (12.7%) compared to males (5.3%) with statistical significance (*p* = 0.037). Other severe toxicities, including weight loss ≥ grade 3 (1.6% vs. 6.9%, *p* = 0.137) and mucositis ≥ grade 3 (4.8% vs. 2.8%, *p* = 0.432), did not differ significantly between sexes. Taste disorders were more prevalent in males (72.4% vs. 60.3%, *p* = 0.063), approaching statistical significance. Less common toxicities, such as hearing loss, osteoradionecrosis, and PEG placement after radiotherapy, were rare and showed no significant sex-based differences. Toxicities are summarized in Table 3.

**Table 3:** Comparison of Treatment-Related toxicities between female and male patients

Toxicity	Overall		
	Female	Male	*p*-value
Fatigue	46 (73.0%)	185 (75.2%)	0.721
Weight loss	39 (61.9%)	149 (60.6%)	0.846
- grade ≥ 3	1 (1.6%)	17(6.9%)	0.137
Dysphagia	54 (85.7%)	196 (79.7%)	0.276
Taste disorders	38 (60.3%)	178 (72.4%)	*0.063*
Xerostomia	41 (65.1%)	167 (67.9%)	0.672
Mucositis	49 (77.8%) 3 (4.8%)	171 (69.5%) 7 (2.8%)	0.196 0.432
Dermatitis	60 (95.2%)	224 (91.1%)	0.278
- grade ≥ 3	8 (12.7%)	13 (5.3%)	**0.037**
Skin hyperpigmentation	17 (27.0%)	60 (24.4%)	0.671
Lymphedema	8 (12.7%)	56 (22.8%)	*0.079*
Hearing loss	1 (1.6%)	6 (2.4%)	> 0.999
Osteoradionecrosis	1 (1.6%)	7 (2.8%)	> 0.999
PEG after RT	1 (1.6%)	10 (4.1%)	0.472

### Local control

**Fig. 1:**
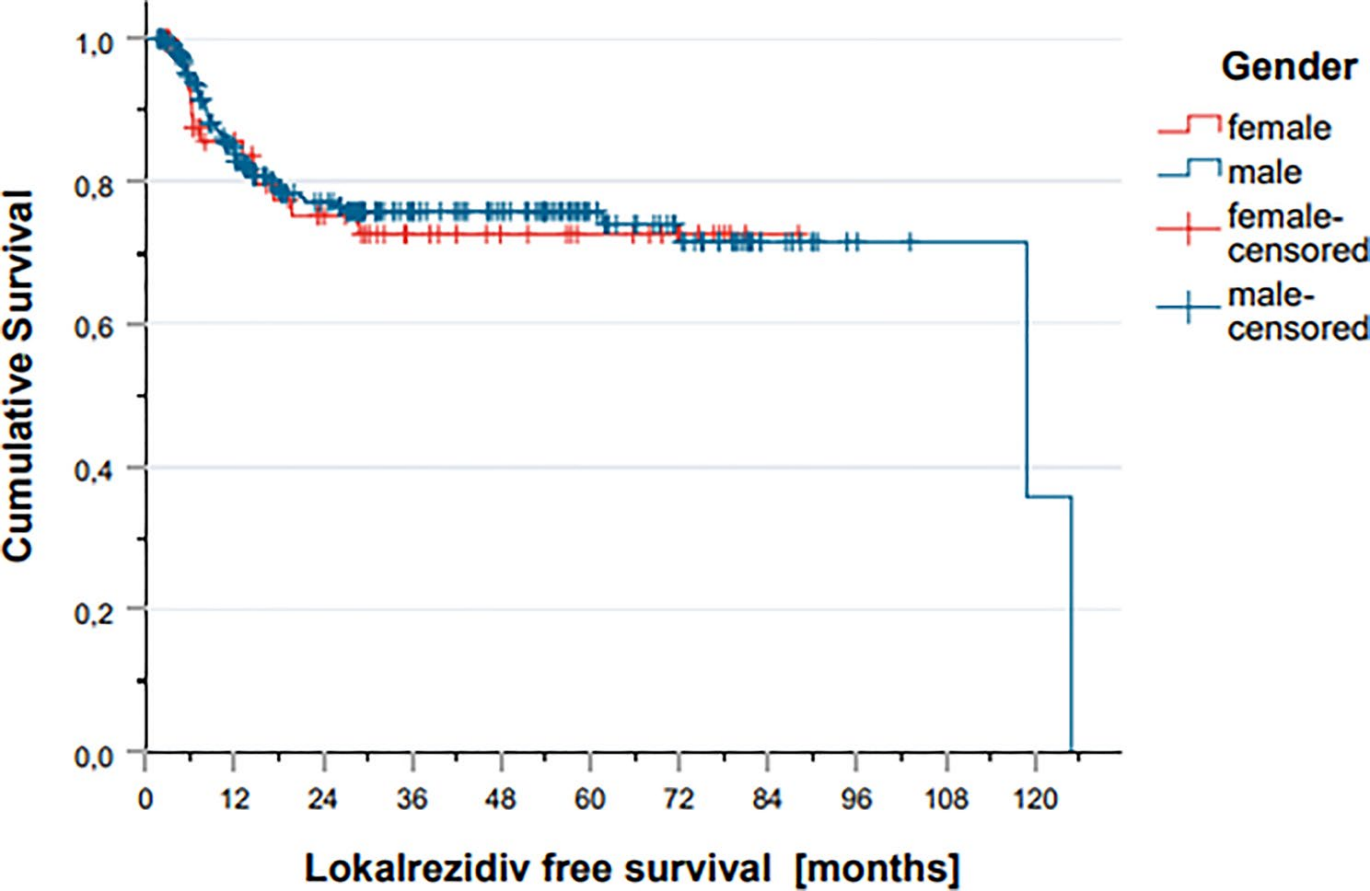
Local recurrence events across the entire cohort and between male and female patients

Local recurrence events were similarly distributed across the entire cohort and between treatment settings (definitive vs. adjuvant), with no statistically significant differences observed between male and female patients overall (Fig. 1). However, pattern A nodal failures—central recurrences within the high-dose nodal volume—were significantly more common in females (64.3%) compared to males (28.8%; *p* = 0.014). No significant sex-based differences were found in other recurrence patterns, including pattern A at the primary site (50.0% in females vs. 60.4% in males), pattern B (7.1% vs. 15.1%), pattern C (21.4% vs. 30.2%), pattern D (7.1% vs. 9.6%), and pattern E (7.1% vs. 15.1%). A summary of recurrence patterns is provided in Table 4.

**Table 4:** Patterns of local and regional recurrence in female and male patients

Patterns of relapse			
	Female *n* = 14	Male *n* = 53	*p*-value
Group A (central high dose volume)			
- *1 (primary tumor)*	7 (50%)	32 (60.4%)	0.484
- *2 (positive nodes)*	9 (64.3%)	15 (28.8%)	***0.014***
***Group B (marginal high dose volume)***			
- *1 (primary tumor)*	1 (7.1%)	8 (15.1%)	0.672
- *2 (positive nodes)*	1 (7.1%)	8 (15.1%)	0.672
***Group C (central elective volume)***	3 (21.4%)	16 (30.2%)	0.741
***Group D (marginal elective volume)***	1 (7.1%)	5 (9.6%)	> 0.999
***Group E (outside of RT volume in head and neck region)***	1 (7.1%)	8 (15.1%)	0.672

Comparison of local recurrence between sexes, stratified by key variables including tumor site, HPV status, p16-status, T and N stage, systemic therapy, and occurrence of distant metastases showed no statistically significant differences.

### Survivals

In the adjuvant RT setting, females exhibited a trend toward better long-term OS, with higher cumulative survival beyond 100 months. In contrast, males in the definitive RT group showed slightly improved OS after 40 months. However, these differences were not statistically significant.

Across the full cohort, no significant differences in OS were observed between male and female patients. Comparison of OS between sexes, stratified by key variables including tumor site, HPV status, p16-status, T and N stage, systemic therapy, occurrence of local or distant recurrence, and patterns of local failure revealed no statistically significant differences. In both radiotherapy subgroups, survival curves remained closely aligned throughout the follow-up period, and sex did not emerge as a significant predictor of OS in either treatment cohort.

**Fig. 2:**
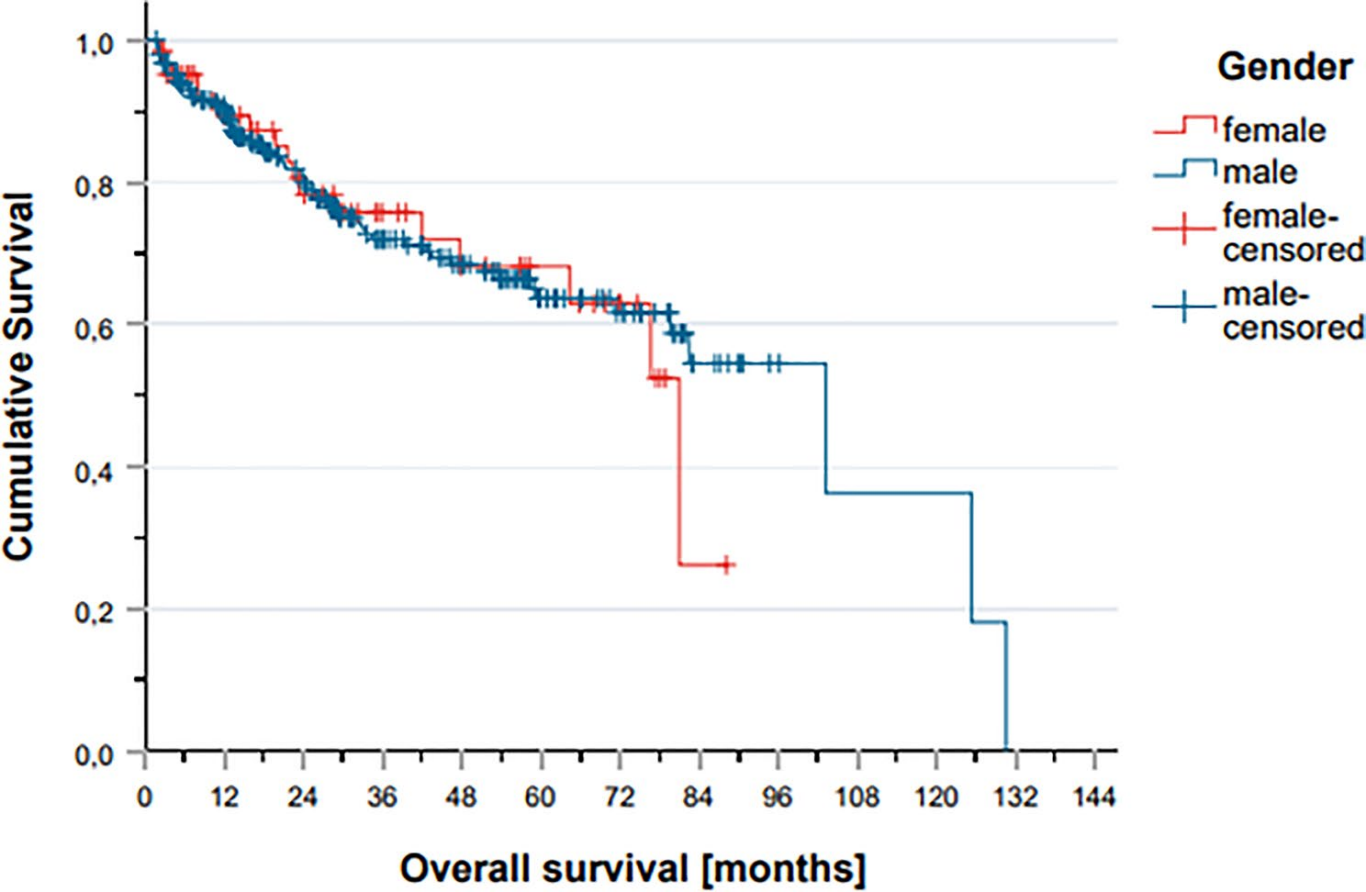
Overall survival events across the entire cohort and between male and female patients

Also, PFS and MFS were similar between sexes across all groups. In the adjuvant subgroup, females showed a non-significant numerical advantage in PFS over time. However, in both the adjuvant and definitive RT settings, survival curves remained largely overlapping, and no statistically significant sex-based differences in disease progression were identified. Comparison of genders stratified according to prognostic factors such as Figure 2a and b, and 2c illustrate these findings.

## Discussion

**Fig. 3:**
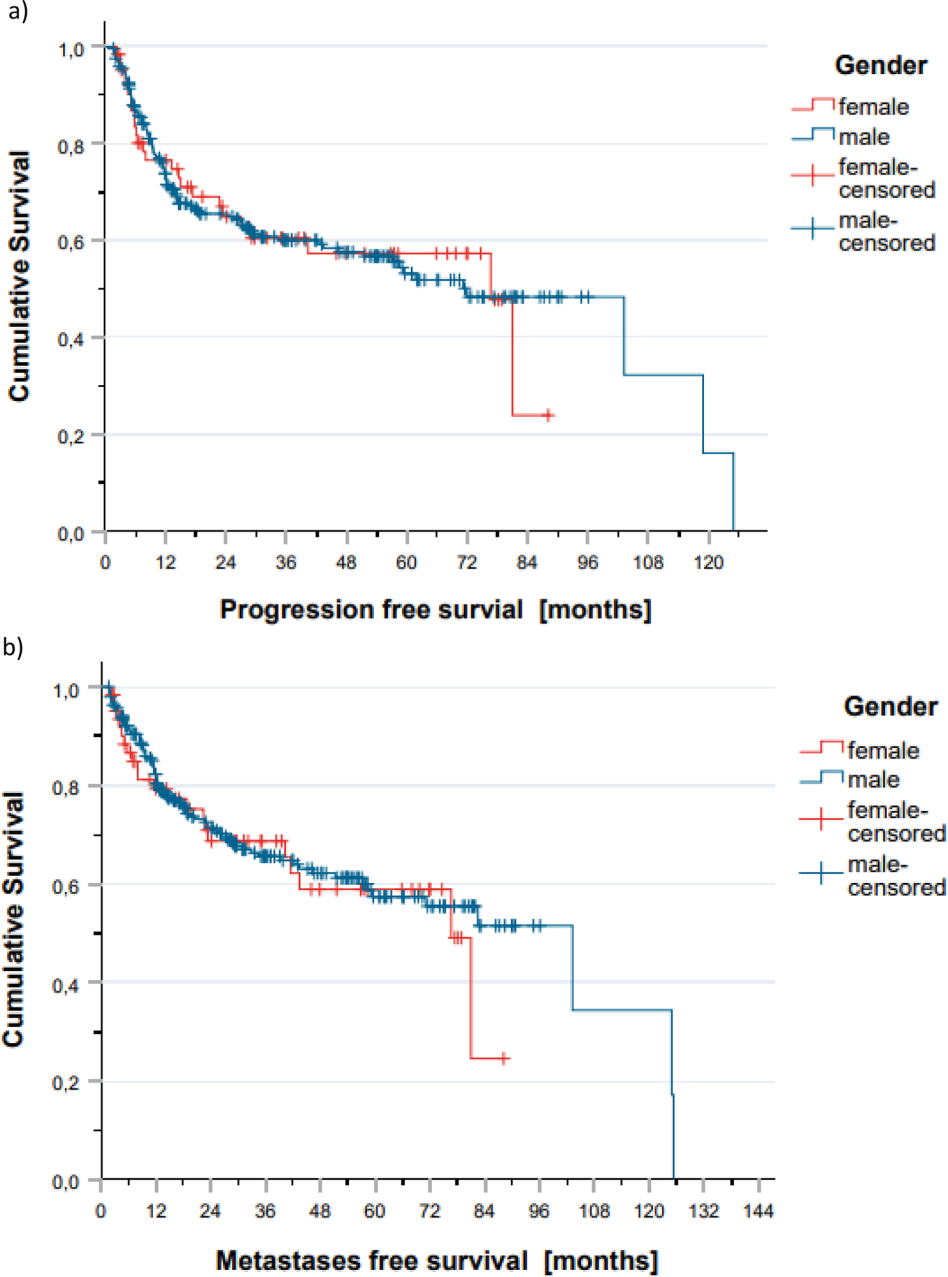
Progression free survival (**a**) and metastases free survival (**b**) events across the entire cohort and between male and female patients

In this retrospective, single-institution cohort study comprising 309 patients with locally advanced SCC of the pharynx and larynx treated with curative-intent radiotherapy, no statistically significant differences in survival outcomes were observed between female and male patients. The study population was well-balanced with respect to patient demographics, tumor characteristics, and treatment modalities, with no significant sex-based disparities in clinical staging, HPV status, or systemic therapy administration. Analysis of ontologically relevant endpoints demonstrated comparable OS, PFS), and MFS between sexes in both definitive and postoperative treatment settings. Furthermore, univariate analysis did not reveal any patient- or tumor-specific variables that were significantly associated with sex in relation to survival outcomes (Fig. 3).

These findings are partially consistent with prior population-based and registry studies. For instance, a retrospective study in a large German cancer registry (1996–2016) found that male gender was an independent and significant risk factors for worse OS and that gender has an influence on incidence per age group and tumor subsite, and on treatment decision, especially in advanced stage and elderly HNC patients [13]. On the other hand, Koffler et al. reported no significant difference in survival between female and male patients in a very recent Austrian large retrospective cohort on 1115 patients after adjustment for clinical covariates. In addition, a recent analysis of the U.S. National Cancer Database focusing on oropharyngeal SCC revealed no independent survival advantage for female patients comprising 19% of the population, but they were more likely to present with HPV-negative disease, earlier-stage tumors, and greater comorbidity burden, and experienced lower survival in the HPV-negative subgroup (HR = 1.11, *p* < 0.001), but not in HPV-positive cases [14]. Our results showed no difference in survival in HPV-positive and negative cases, respectively, between male and female (data not shown), although the females more HPV pos -… The prognostic relevance of biological sex in HNSCC remains debated, with prior studies yielding inconsistent results across tumor subsites and HPV status. Overall, current evidence indicates that biological sex, when isolated from confounding factors, is not a consistent or independent determinant of survival in patients receiving curative-intent radiotherapy for locally advanced HNSCC.

Despite overlapping survival curves, we observed a higher incidence of grade ≥ 3 dermatitis among female patients, consistent with findings by Pilśniak et al., who reported that women more frequently exhibited dermoscopic signs of chronic radiation-induced dermatitis—particularly pronounced vascular features such as linear and dotted vessels [15]. These results suggest a potential sex-related predisposition to late cutaneous radiation toxicity in head and neck cancer and support the hypothesis of increased radiation sensitivity in females [16]. Preclinical evidence indicates that sex steroid hormones modulate DNA repair pathways, with estrogen shown to influence homologous recombination and non-homologous end joining, and androgens enhancing DNA-dependent protein kinase–mediated repair. These findings suggest that biological sex, through hormone-regulated DNA damage response mechanisms, may significantly impact radiosensitivity and treatment outcomes in cancer patients particularly in skin and mucosal tissues [17, 18]. Despite growing experimental evidence elucidating how sex-defining factors such as chromosomes and gonadal hormones modulate immune responses, the mechanisms linking sex differences to immune-mediated conditions remain incompletely understood, underscoring the importance of this knowledge for advancing targeted patient care [19].

A novel finding in our study was the significantly higher rate of pattern A nodal failures (central high-dose nodal recurrence) in female patients. This unexpected result may point to subtle sex-based biological or dosimetric divergences. While other molecular and treatment-related studies have underscored the importance of precise nodal dosing and immunophenotypic differences in women [20], none have specifically associated sex with in-field nodal relapse in HNSCC. This suggests potential sex-related variations in radiation response or microenvironmental factors, warranting further prospective investigation.

Strengths of this study include the uniform institutional RT protocols employing IMRT/VMAT with consistent delineation and CBCT guidance, comprehensive toxicity and recurrence assessments, and detailed classification of failure patterns. Additionally, centralized pathological and radiological review strengthens the internal validity.

Nevertheless, several limitations must be acknowledged. The retrospective design inherently introduces selection and information bias. The sample size, particularly of female patients and recurrence events, may be insufficient to detect modest but clinically relevant sex-related differences. Hormonal status and gender identity, which may influence both biology and treatment tolerance, were not recorded. Finally, dosimetric parameters—such as exact dose coverage and radiobiological modeling—were not analyzed, which could further explain the observed sex-specific differences in nodal failure.

## Conclusion

Our data indicate that sex does not independently predict survival or disease control in curatively treated locally advanced HNSCC. The increased toxicity and in-field nodal recurrence observed in female patients highlight the need for prospective evaluation of sex-specific treatment adaptation, supportive care optimization, and radiobiological research to refine personalized RT strategies.

## Supplementary Information

Below is the link to the electronic supplementary material.

Supplementary Material 1: Figure 1: Local recurrence events across male and female patients in the adjuvant (a) and definitive (b) treatment setting between male and female patients. Figure 2: Overall survival events across male and female patients in the adjuvant (a) and definitive (b) treatment setting between male and female patients. Figure 3: Progression free survival events across male and female patients in the adjuvant (a) and definitive (b) treatment setting between male and female patients. Figure 4: Metastases free survival Local recurrence events across male and female patients in the adjuvant (a) and definitive (b) treatment setting between male and female patients.

## Data Availability

No datasets were generated or analysed during the current study.
