# Supplementary material for: Sex-based differences in patients with locally advanced pharyngeal and laryngeal SCC treated with definitive or adjuvant radiotherapy
Source: Radiat Oncol. 2025 Dec 4;20:184. doi: 10.1186/s13014-025-02771-z (PMC12690892; doi:10.1186/s13014-025-02771-z)

Figure 1: Local recurrence events across male and female patients in the adjuvant (a) and definitive (b) treatment setting between male and female patients.

a)


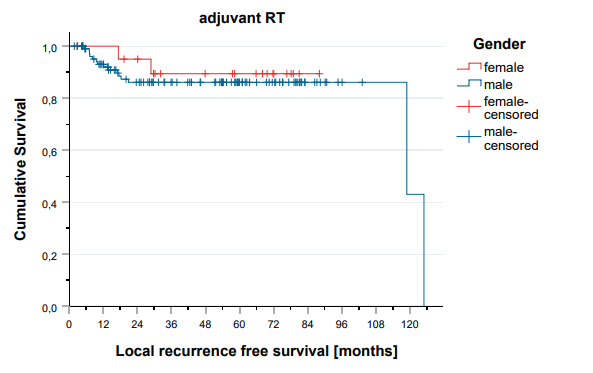


b)


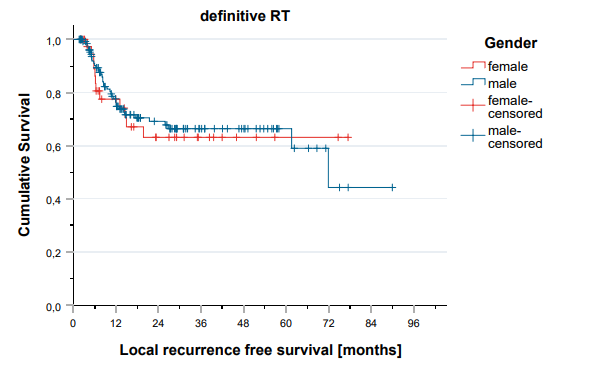


Figure 2: Overall survival events across male and female patients in the adjuvant (a) and definitive (b) treatment setting between male and female patients.

a)


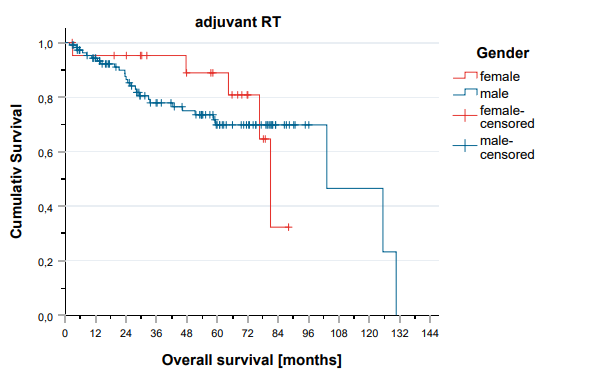


b)


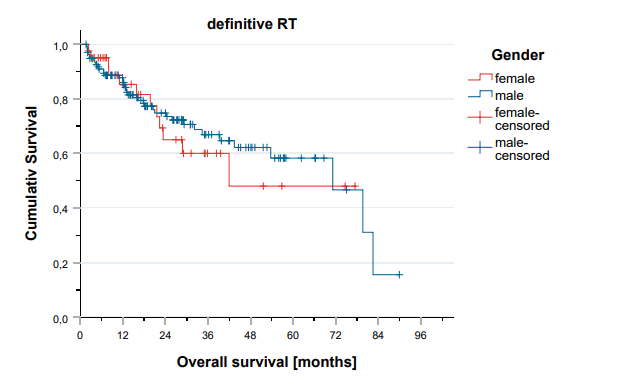


Figure 3: Progression free survival events across male and female patients in the adjuvant (a) and definitive (b) treatment setting between male and female patients.

a)


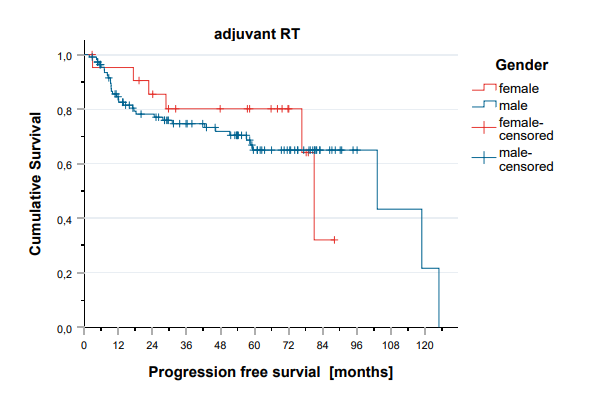


b)


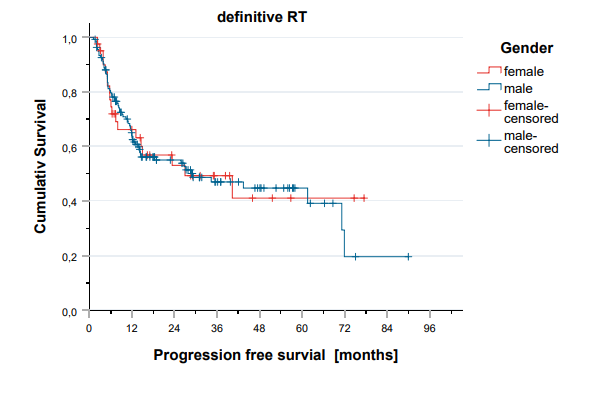


Figure 4: Metastases free survival Local recurrence events across male and female patients in the adjuvant (a) and definitive (b) treatment setting between male and female patients.

a)


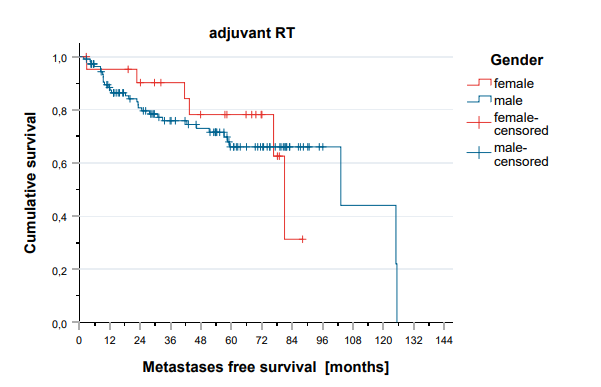


b)


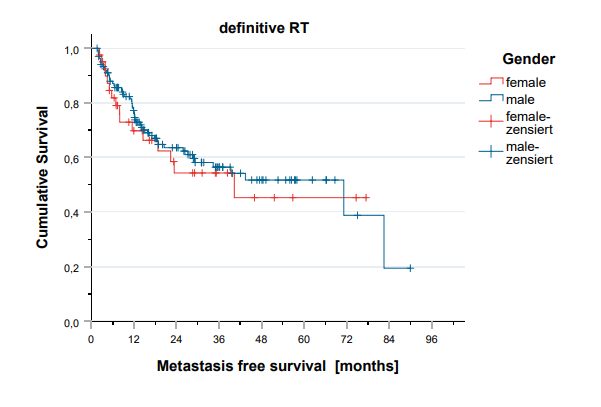

Supplement: Supplementary file 1 — Supplementary Material 1: Figure 1: Local recurrence events across male and female patients in the adjuvant (a) and definitive (b) treatment setting between male and female patients. Figure 2: Overall survival events across male and female patients in the adjuvant (a) and definitive (b) treatment setting between male and female patients. Figure 3: Progression free survival events across male and female patients in the adjuvant (a) and definitive (b) treatment setting between male and female patients. Figure 4: Metastases free survival Local recurrence events across male and female patients in the adjuvant (a) and definitive (b) treatment setting between male and female patients. [file 13014_2025_2771_MOESM1_ESM.docx]
